# Identification of a novel botulinum neurotoxin gene cluster in *Enterococcus*

**DOI:** 10.1002/1873-3468.12969

**Published:** 2018-01-23

**Authors:** Jason Brunt, Andrew T. Carter, Sandra C. Stringer, Michael W. Peck

**Affiliations:** ^1^ Gut Health and Food Safety Quadram Institute Norwich UK

**Keywords:** botulinum neurotoxin, *Clostridium*, eBoNT/J, *Enterococcus*

## Abstract

The deadly neurotoxins of *Clostridium botulinum* (BoNTs) comprise eight serotypes (A–G; X). The neurotoxin gene cluster encoding BoNT and its accessory proteins includes an operon containing an *ntnh* gene upstream of the *boNT* gene. Another operon contains either *ha* (haemagglutinin) or *orfX* genes (of unknown function). Here we describe a novel *boNT* gene cluster from *Enterococcus* sp. 3G1_DIV0629, with a typical *ntnh* gene and an uncommon *orfX* arrangement. The neurotoxin (designated putative eBoNT/J) contains a metallopeptidase zinc‐binding site, a translocation domain and a target cell attachment domain. Structural properties of the latter suggest a novel targeting mechanism with consequent implications for application by the pharmaceutical industry. This is the first complete *boNT* gene cluster identified in a non‐clostridial genome.

## Abbreviations

**GI**, genomic island

**HA**, haemagglutinin

**NCBI**, National Center for Biotechnology Information

**wgMLST**, whole genome multilocus sequence typing

**WGS**, whole genome sequence

Neurotoxins produced by *Clostridium botulinum* and occasional strains of *C. baratii* and *C. butyricum* (BoNTs) cause a severe and fatal neuro‐paralytic disease of humans and animals (botulism) 1. Currently there are eight recognised serotypes of the BoNT protein (A–G, and recently X 2, 3). Biologically active BoNT is complexed with several accessory proteins, all encoded by a neurotoxin gene cluster. This gene cluster is often associated with mobile elements or is located on a plasmid or bacteriophage, indicating that it is capable of horizontal gene transfer between bacteria sharing a common environment 4. The gene for the coexpressed protein NTNH is always located upstream of the gene that encodes BoNT. A further operon encodes typically three genes that fall into two categories; *ha* genes (haemagglutinin) and *orfX* genes (of unknown function) 2. Accessory gene products are needed to ensure survival of the BoNT toxin complex during its passage through the gastrointestinal tract, and for transfer through the gut wall into the circulatory system with subsequent delivery to the target nerve cell, although the exact mechanism for translocation across the gut epithelium has only been shown for the HA proteins 2, 5. BoNT is the most potent toxin known 1. It is a zinc metallopeptidase with an extreme specificity for its target, the SNARE docking proteins of cholinergic nerve cells. BoNT activity destroys the function of these SNAREs, preventing exocytosis of the neurotransmitter acetylcholine with subsequent floppy paralysis of associated muscle tissue 6. BoNT is used both in the cosmetic and pharmaceutical industries. As such, there is great interest in the discovery of new forms of BoNT, in the hope that these will increase the range of medical conditions that can be alleviated.

Here we describe the discovery of a novel *boNT* gene cluster that exists not in the *C. botulinum*,* C. baratii* or *C. butyricum* genome but within the genome of a species of *Enterococcus*. The *Enterococcus* sp. 3G1_DIV0629 genome contains a botulinum‐like neurotoxin gene cluster with a typical *ntnh* gene and an uncommon *orfX* arrangement. The predicted neurotoxin gene product from this cluster (designated herein putative eBoNT/J) contains all the functional domains characteristic of a typical neurotoxin, including a metallopeptidase zinc‐binding site, a translocation domain and a target cell attachment domain 7. Structural properties of the latter domain suggest a novel targeting mechanism with consequent implications for application by the pharmaceutical industry. This the first report of a complete new botulinum‐like neurotoxin gene cluster outside of the *Clostridium* species.

## Methods

Putative eBoNT/J was identified using known BoNT protein sequences to search the whole genome sequence (WGS) database (visited October 2017) at the National Center for Biotechnology Information (NCBI). An unrooted NeighborNet phylogenetic network of clostridial neurotoxin proteins was computed using the SplitsTree4 application 8. Parameters used for preliminary sequence alignment in Geneious 9 using clustalw were: cost matrix blosum; gap open cost 10; gap extend cost 0.1. The programme simplot 10 from the dambe software suite 11 was used to compare putative eBoNT/J and its associated NTNH with representative examples of other neurotoxin amino acid sequences; we performed this analysis to identify possible mosaic sequences. Parameters used were: window size 100, step length 20, genetic distance PoissonP, using either putative eBoNT/J or NTNH as seed. An iterative search method (JACKHMMER) was used to compare the predicted gene product of putative eBoNT/J with reference proteomes of *C. botulinum* until convergence. Functional protein domains were identified using HMMER 12 and Pfam 13.

To determine the closest relative of strain *Enterococcus* sp. 3G1_DIV0629, we used the web‐based tool PGAdb‐builder, which uses a pipeline based on whole genome multilocus sequence typing (wgMLST; 14). Genome sequences were downloaded in FASTA file format as contigs or complete genome sequences from the NCBI website. For phylogenetic analysis, representative enterococcal genome sequences 15 were reannotated using Prokka 16, and comparative genomics performed using Roary 17. The phylogenetic tree was produced (UPGMA) with the phylip programme using the constructed allelic sequences and bootstrap values calculated by the ETE tool 18. DNA G + C content of complete genomes was taken from the appropriate NCBI Genome Assembly and Annotation report pages. islandviewer 4 19 was used for identification and visualisation of genomic islands (GIs).

The amino acid sequence of putative eBoNT/J was further analysed using the programme Phyre2 20, which predicts 3D protein structure. Further analysis and comparison of putative eBoNT/J toxin and its associated NTNH to BoNT/A and BoNT/A complexed with its NTNH was performed by using the I‐Tasser simulation 21 which selects the best structural model that fits the query sequence. Estimated accuracy of the predicted model using I‐TASSER was; TM score 0.86 ± 0.07, *C*‐score = 1.07 and RMSD = 7.0 ± 4.1 Å (eBoNT/J) and TM score 0.87 ± 0.07, *C*‐score = 1.17 and RMSD = 6.6 ± 4.0 Å (NTNH).

## Results and Discussion

**Figure 1:**
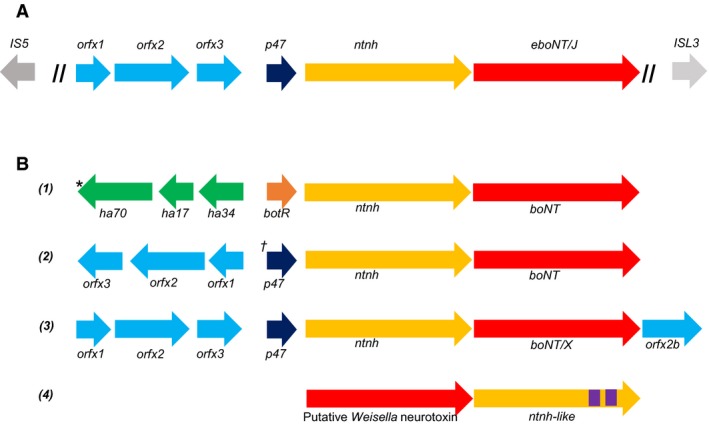
Neurotoxin gene clusters of (A) *Enterococcus* sp. 3G1_DIV0629; (B1) *C. botulinum* Group I *ha* types A, B; *C. botulinum* Group II type B; *C. botulinum* Group III (*Group III neurotoxin gene clusters position the gene for transcription factor *botR* upstream of ha70); *C. botulinum* Group IV (the type G neurotoxin gene cluster swaps the positions of *ha70* and *ha34*). (B2) Group I *orf‐X* types A, F († Group I neurotoxin gene clusters include *botR* at this position); Group II types E, F. (B3) *boNT/X*. (B4) *Weissella oryzae SG25*. Purple regions in *ntnh*‐like refer to Big 3 domains that are not present in the *ntnh*‐like gene. Note that the putative *Weisella* neurotoxin gene is not associated with *ntnh*,* ha* or *orfx* accessory genes.

A bioinformatics search of the WGS database (visited October 2017) at the NCBI, using the predicted protein translation product of *boNT* genes scored a hit with the product of a gene from a recently deposited (Earl A. *et al*. May 2017) genome of *Enterococcus* sp. 3G1_DIV0629 (NCBI accession number NGLI01000004.1; this refers to contig 4 from the sequencing assembly). The *Enterococcus* putative neurotoxin gene product shared 39% identical residues with its closest relative, BoNT/X, with 58% residues exhibiting conservative changes. The contig containing the *boNT*‐like gene was further examined, to reveal a set of genes upstream that were similar to the *orfX1*,* orfX2*,* orfX3*,* p47* and *ntnh* genes of other *orfX*‐type *boNT* neurotoxin gene clusters 2, 3 although distantly related (26–36% amino acid sequence to closest relative, BoNT/X). In all other examples of *orfX* neurotoxin gene clusters, only the *p47* gene respects the direction of expression of *ntnh* and *boNT*, with the three *orfX* genes facing in the opposite direction (Fig. 1). Although the recently discovered botulinum neurotoxin homologue in *Weissella oryzae* SG25 22 has been tentatively named *boNT/Wo* 23, until neurotoxicity studies have been performed, we propose to call this new homologue putative *eboNT/J*. As with many *boNT* gene clusters, that of putative eBoNT/J is bordered by IS elements 2, 24, evidence that it may have been acquired by horizontal gene transfer (Fig. 1).

**Figure 2:**
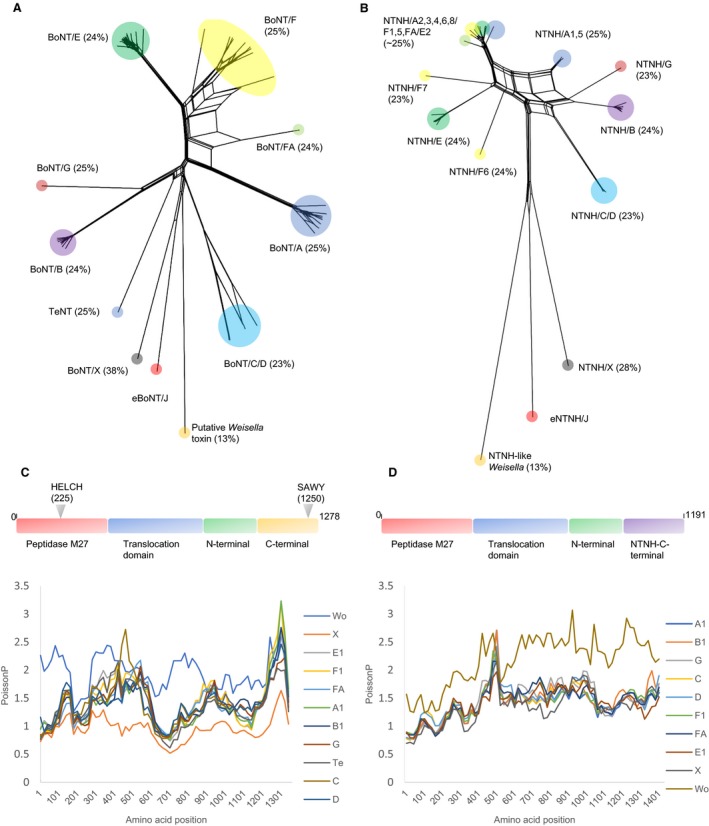
(A,B) SplitsTree plots of BoNT and NTNH protein sequences respectively. TeNT protein is not associated with an NTNH accessory protein. Both plots show that BoNT/X and eBoNT/J and their associated NTNH are more closely related to each other than they are to all other proteins. (C,D). SimPlot analyses of BoNT and NTNH protein domain sequences, respectively, using eBoNT/J and its NTNH as seeds; main functional domains of each eBoNT/J cluster protein are outlined above. The closer relatedness of BoNT/X to eBoNT/J continues throughout its length; however, the NTNH associated with eBoNT/J is almost equally distant from all other examples.

A NeighborNet phylogenetic network of clostridial neurotoxin proteins (including the putative neurotoxin of *W. oryzae* SG25) was estimated using the splitstree programme. The output can be compared directly with that used to demonstrate the discovery of BoNT/X 3. Predicted gene products of the adjacent *ntnh* gene were similarly analysed (Fig. 2A,B). As shown by the position and length of their branchpoint, putative eBoNT/J is most closely related (38% identity) to BoNT/X, and all other neurotoxins are equally distant (23–25% identity), apart from the putative neurotoxin of *Weisella* which shares the least protein identity (13%). A similar result was obtained with the putative eBoNT/J NTNH protein.

An iterative search (JACKHMMER) was used to compare the predicted gene product of putative eBoNT/J with reference proteomes of *C. botulinum* until convergence. The resulting 232 matches to botulinum toxin showed that putative eBoNT/J possesses all domains known to be required for BoNT activity; furthermore, these are located in their correct positions 7 (Fig. 2C). These include a light chain containing the zinc binding site of an M27 peptidase (HELCH) at positions 225–229 25, cysteine residues at positions 424 and 438 required for the disulphide bridge between heavy and light chains following proteolytic cleavage and activation 7 (putative eBoNT/J lacks the extra C residue in this linker region that is present in BoNT/X), a translocation domain (residues 529–843) at the N terminus of the heavy chain containing a version (PYLGNIL, residues 622–628; in BoNT/X this is PYIGPLL) of the conserved PYxGxAL motif required for toxin translocation from the endosome into the target nerve cell cytoplasm 26 and N and C termini (H_CN_, H_CC_) of the C‐terminal‐binding domain of the heavy chain that are required for binding to the target cell and initiation of endocytosis. With BoNT/A, B, E, F and G, this appropriation of the normal host synaptic vesicle recycling pathways involves a dual host–receptor mechanism comprising a synaptic vesicle protein and a ganglioside 27, 28, 29, 30, 31. These BoNT–ganglioside interactions are facilitated by a SxWY motif located in the C terminus of the heavy chain binding domain; in putative eBoNT/J, this motif is SAWY (residues 1250–1253) and is identical to that of BoNT/X.

Similarity plots were used to further analyse putative eBoNT/J and its accompanying NTNH for relatedness to other BoNT and NTNH proteins (Fig. 2C,D). As indicated by SplitsTree, BoNT/X remained the closest relative throughout its entire length (Fig. 2C); however, all NTNH sequences seemed to be approximately equally distant from that of putative eBoNT/J (Fig. 2D), except for the NTNH‐like peptide associated with the putative neurotoxin of *Weisella* which is a clear outlier.

**Figure 3:**
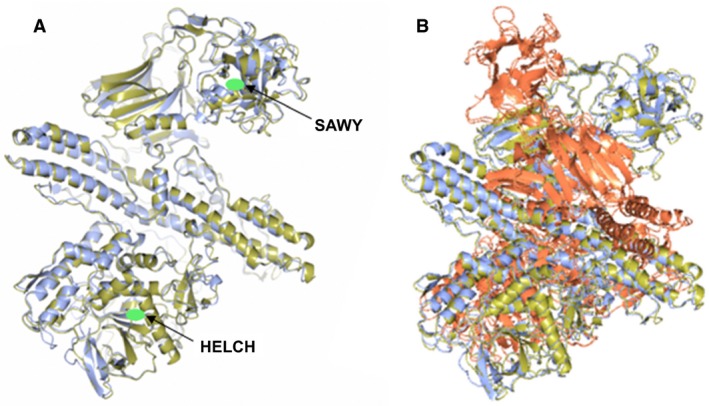
Structure prediction and comparison of putative eBoNT/J toxin and its associated NTNH to BoNT/A and BoNT/A complexed with its NTNH, respectively.(A) Superposition of the predicted structure of putative eBoNT/J (gold) with the crystal structure of an inactive BoNT/A (blue Protein Data bank ID: 3V0C). Positions of the zinc‐binding site (HELCH) and target cell‐binding motif (SAWY) are indicated by green ovals. (B) Superposition of the predicted structure of the eBoNT/J NTNH (gold) with that determined for BoNT/A (red) complexed with its own NTNH (blue; Protein Data bank ID: 3V0B).

The amino acid sequence of putative eBoNT/J was further analysed using the programme Phyre2 20, which predicts 3D protein structure. This predicted a protein structure for putative eBoNT/J that exactly matched a BoNT with a 100% confidence limit. This shows that not only does the entire length of putative eBoNT/J share amino acid sequence conservation with other BoNTs but it also shares structural identity. Another modelling programme, I‐TASSER showed that the predicted structure for putative eBoNT/J most closely matched that of a BoNT/A molecule; this match was also mirrored when the NTNH associated with eBoNT/J was superimposed with the structure determined for BoNT/A complexed with its own NTNH, suggesting that if expressed, the putative eBoNT/J could form a similar complex (Fig. 3).

Electron microscopy studies of progenitor BoNT/A complex have shown that its NTNH moiety carries the binding site for HA70, occupied during formation of the active botulinum toxin complex. This binding site is conserved in other BoNTs that form complexes with HA moieties. Those BoNTs that derive from an *orf‐X* neurotoxin gene cluster lack this site, which has been located to a 33‐residue region located ~ 120 residues from the N terminus, termed the nLoop 32, 33. Both BoNT/X and putative eBoNT/J share this deletion, which is evidence that they have evolved alongside their Orf‐X accessory proteins.

**Figure 4:**
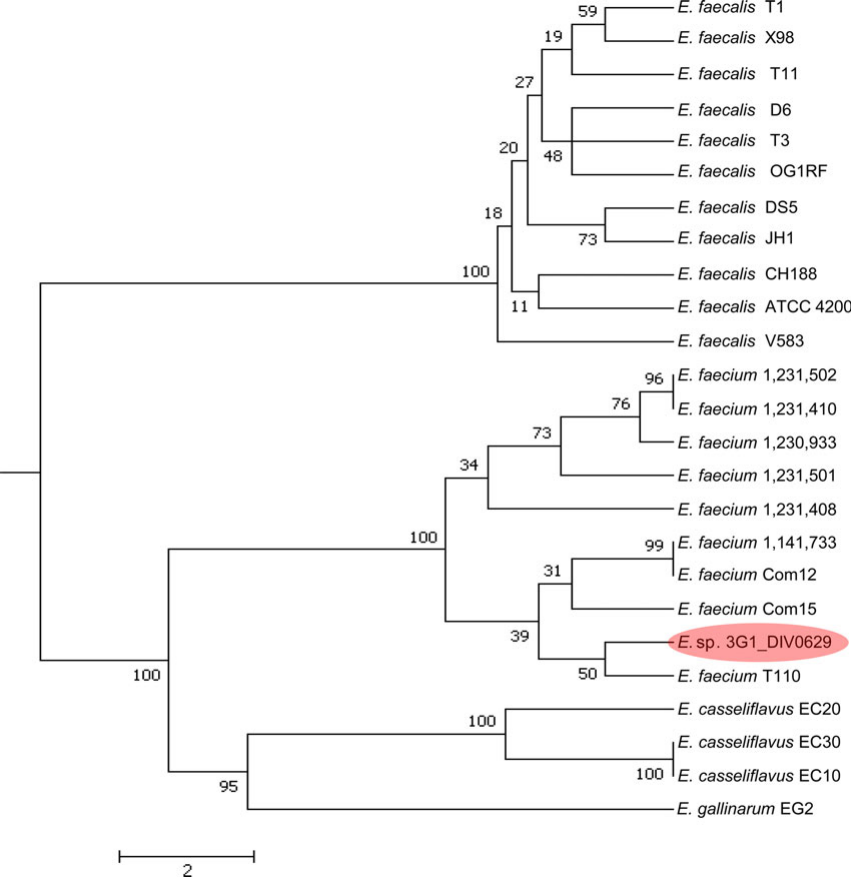
Phylogenetic tree of *Enterococcus,* showing close relatedness of *Enterococcus* sp 3G1_DIV0629 to *E. faecium* T110.

Species of *Enterococcus* are Gram‐positive bacteria of the phylum Firmicutes, order Lactobacillales that can be commensal in the gastrointestinal tract of humans and animals, but may also be pathogenic, causing diseases such as neonatal meningitis or endocarditis 34. *Enterococcus* sp. 3G1_DIV0629 was isolated from cow faeces in South Carolina, USA. It is distinct from currently recognised species, so has not yet been given a specific name (see NCBI project number PRJNA313452). To determine the closest relative of this strain, we used wgMLST to establish that *Enterococcus* sp. 3G1_DIV0629 was most closely related to the probiotic strain *Enterococcus faecium* T‐110 35, (Fig. 4). A search of all publicly available (> 1000) *Enterococcus* genomes failed to identify another strain containing a botulinum neurotoxin gene cluster.

To determine whether the putative *eboNT/J* neurotoxin gene cluster has been acquired via horizontal gene transfer, its DNA G + C content was analysed. The G + C content of all sequenced genomes of *E. faecium* ranges between 36.7% and 42.8%; that of a group of 77 uncharacterised isolates of *Enterococcus* (of which strain 3G1_DIV0629 is a member) is 33.1–43.3%. The average G + C content of the *Enterococcus* sp. 3G1_DIV0629 genome is 37.2%, which falls within these ranges. However, contig 4, which contains the putative *eboNT/J* neurotoxin gene cluster has an unusually low G + C content (31.8% as compared to 38.0–39.3% for the other main (> 50 kb) contigs). That for a 20 kb region of contig 4 encompassing the putative *eboNT/J* neurotoxin gene cluster, including the IS elements upstream and downstream, was 31.8%, which is identical to the rest of the same contig. Using the DNA sequence of contig 4 as a query, the NCBI WGS database of all sequenced members of phylum Firmicutes was interrogated. All regions of contig 4 which generated a match (~ 30%) were to enterococcal plasmid sequences; particularly to an ~ 52 kb region in the centre of the contig, mapping immediately upstream of the neurotoxin gene cluster. However, isolated matches to enterococcal plasmid sequences were scattered throughout contig 4. Considering that the size of contig 4 matches that of several examples of enterococcal plasmids, it is possible that this entire contig represents a plasmid sequence. However, it is equally possible that contig 4 represents a horizontally acquired GI, the G + C content of which is often typically lower than that of its host genome 36. Using islandviewer 4, a software programme for identification and visualisation of GIs 19, a comparison between *Enterococcus* sp. 3G1_DIV0629 and its closest relative *E. faecium* T‐110 shows that the putative *eboNT/J* toxin gene cluster lies between two predicted pathogenicity islands (a type of GI); however, comparison of the G + C plot of this region with that of the entire chromosome of strain T‐110 indicates that the entire contig may be a GI (data not shown). More sequencing work is needed to confirm this speculation, as the insertion sites for GIs are often found at the switch sites of GC‐skew 37. The DNA G + C content of 203 sequenced *C. botulinum* genomes in the NCBI genome database exhibits a narrow range of 27.0–29.8%, somewhat lower than that for *Enterococcus*. This probably reflects the fact that the last common ancestor of the Lactobacillales and the Clostridiales existed ~ 2.8 billion years ago 38 and suggests that if the putative *eboNT/J* neurotoxin gene cluster has been acquired horizontally (as suggested by the presence of the two IS elements, Fig. 1), then the donor organism may not be *C. botulinum*.

## Conclusion

In summary, this work reports the bioinformatic discovery of the first complete *boNT* toxin gene cluster located in a non‐clostridial genome. The organisation and sequence identity of this gene cluster shows that its closest relative is the recently published *boNT/X* cluster from *C. botulinum* strain 111. Although amino acid sequence homology with BoNT/X is only 38%, 3D structure modelling shows that putative eBoNT/J closely mimics the structure of the most potent neurotoxin, BoNT/A. Significantly, as with BoNT/X, variation in the relevant region of the C terminus of the heavy chain indicates that it may possess a novel cell‐binding domain. Further work will be required to investigate whether this structural variation will have important implications for the potential use of putative eBoNT/J as a therapeutic agent. Finally, as this work is purely a bioinformatics study, with no access to the bacterial strain, there is no information available regarding whether the putative *eboNT/J* toxin gene cluster is expressed by its host. However, the fact that all open reading frames for the putative toxin cluster genes are intact strongly suggests that expression is likely. Associated metadata with the genome sequence do not indicate that the herd from which the faecal sample was taken had suffered from symptoms of botulism. Both questions are intriguing and will be the subject of future work.

## Author contributions

JB and ATC identified putative eBoNT/J and performed the experiments. All authors contributed to data analysis and preparation of the paper. All authors read and approved the final manuscript.

## References

[1] Peck MW (2009) Biology and genomic analysis of *Clostridium botulinum* . Adv Microb Physiol 55, 183–265, 320. 19573697 10.1016/S0065-2911(09)05503-9

[2] Carter AT and Peck MW (2015) Genomes, neurotoxins and biology of *Clostridium botulinum* Group I and Group II. Res Microbiol 166, 303–317. 25445012 10.1016/j.resmic.2014.10.010 PMC4430135

[3] Zhang S , Masuyer G , Zhang J , Shen Y , Lundin D , Henriksson L , Miyashita SI , Martinez‐Carranza M , Dong M and Stenmark P (2017) Identification and characterization of a novel botulinum neurotoxin. Nat Commun 8, 14130. 28770820 10.1038/ncomms14130 PMC5543303

[4] Hill KK , Xie G , Foley BT and Smith TJ (2015) Genetic diversity within the botulinum neurotoxin‐producing bacteria and their neurotoxins. Toxicon 107, 2–8. 26368006 10.1016/j.toxicon.2015.09.011

[5] Lam KH and Jin R (2015) Architecture of the *botulinum* neurotoxin complex: a molecular machine for protection and delivery. Curr Opin Struct Biol 31, 89–95. 25889616 10.1016/j.sbi.2015.03.013 PMC4476938

[6] Poulain B , Popoff MR and Molgo J (2008) How do the Botulinum Neurotoxins block neurotransmitter release: from botulism to the molecular mechanism of action. Botulinum J 1, 14.

[7] Montal M (2010) Botulinum neurotoxin: a marvel of protein design. Annu Rev Biochem 79, 591–617. 20233039 10.1146/annurev.biochem.051908.125345

[8] Huson DH and Bryant D (2006) Application of phylogenetic networks in evolutionary studies. Mol Biol Evol 23, 254–267. 16221896 10.1093/molbev/msj030

[9] Kearse M , Moir R , Wilson A , Stones‐Havas S , Cheung M , Sturrock S , Buxton S , Cooper A , Markowitz S , Duran C *et al* (2012) Geneious Basic: an integrated and extendable desktop software platform for the organization and analysis of sequence data. Bioinformatics 28, 1647–1649. 22543367 10.1093/bioinformatics/bts199 PMC3371832

[10] Lole KS , Bollinger RC , Paranjape RS , Gadkari D , Kulkarni SS , Novak NG , Ingersoll R , Sheppard HW and Ray SC (1999) Full‐length human immunodeficiency virus type 1 genomes from subtype C‐infected seroconverters in India, with evidence of intersubtype recombination. J Virol 73, 152–160. 9847317 10.1128/jvi.73.1.152-160.1999 PMC103818

[11] Xia X (2017) DAMBE6: new tools for microbial genomics, phylogenetics, and molecular evolution. J Hered 108, 431–437. 28379490 10.1093/jhered/esx033 PMC5434544

[12] Finn RD , Clements J , Arndt W , Miller BL , Wheeler TJ , Schreiber F , Bateman A and Eddy SR (2015) HMMER web server: 2015 update. Nucleic Acids Res 43, W30–W38. 25943547 10.1093/nar/gkv397 PMC4489315

[13] Punta M , Coggill PC , Eberhardt RY , Mistry J , Tate J , Boursnell C , Pang N , Forslund K , Ceric G , Clements J *et al* (2012) The Pfam protein families database. Nucleic Acids Res 40, D290–D301. 22127870 10.1093/nar/gkr1065 PMC3245129

[14] Liu YY , Chiou CS and Chen CC (2016) PGAdb‐builder: a web service tool for creating pan‐genome allele database for molecular fine typing. Sci Rep 6, 36213. 27824078 10.1038/srep36213 PMC5099940

[15] Palmer KL , Godfrey P , Griggs A , Kos VN , Zucker J , Desjardins C , Cerqueira G , Gevers D , Walker S , Wortman J *et al* (2012) Comparative genomics of enterococci: variation in *Enterococcus faecalis*, clade structure in *E. faecium*, and defining characteristics of *E. gallinarum* and *E. casseliflavus* . MBio 3, e00318‐11. 22354958 10.1128/mBio.00318-11 PMC3374389

[16] Seemann T (2014) Prokka: rapid prokaryotic genome annotation. Bioinformatics 30, 2068–2069. 24642063 10.1093/bioinformatics/btu153

[17] Page AJ , Cummins CA , Hunt M , Wong VK , Reuter S , Holden MT , Fookes M , Falush D , Keane JA and Parkhill J (2015) Roary: rapid large‐scale prokaryote pan genome analysis. Bioinformatics 31, 3691–3693. 26198102 10.1093/bioinformatics/btv421 PMC4817141

[18] Huerta‐Cepas J , Serra F and Bork P (2016) ETE 3: reconstruction, analysis, and visualization of phylogenomic data. Mol Biol Evol 33, 1635–1638. 26921390 10.1093/molbev/msw046 PMC4868116

[19] Bertelli C , Laird MR , Williams KP , Simon Fraser University Research Computing Group , Lau BY , Hoad G , Winsor GL and Brinkman FSL (2017) IslandViewer 4: expanded prediction of genomic islands for larger‐scale datasets. Nucleic Acids Res. 45, W30–W35. 28472413 10.1093/nar/gkx343 PMC5570257

[20] Kelley LA , Mezulis S , Yates CM , Wass MN and Sternberg MJ (2015) The Phyre2 web portal for protein modeling, prediction and analysis. Nat Protoc 10, 845–858. 25950237 10.1038/nprot.2015.053 PMC5298202

[21] Yang J , Yan R , Roy A , Xu D , Poisson J and Zhang Y (2015) The I‐TASSER Suite: protein structure and function prediction. Nat Methods 12, 7–8. 25549265 10.1038/nmeth.3213 PMC4428668

[22] Mansfield MJ , Adams JB and Doxey AC (2015) Botulinum neurotoxin homologs in non‐Clostridium species. FEBS Lett 589, 342–348. 25541486 10.1016/j.febslet.2014.12.018

[23] Zornetta I , Azarnia Tehran D , Arrigoni G , Anniballi F , Bano L , Leka O , Zanotti G , Binz T and Montecucco C (2016) The first non Clostridial botulinum‐like toxin cleaves VAMP within the juxtamembrane domain. Sci Rep 6, 30257. 27443638 10.1038/srep30257 PMC4957215

[24] Dineen SS , Bradshaw M and Johnson EA (2003) Neurotoxin gene clusters in *Clostridium botulinum* type A strains: sequence comparison and evolutionary implications. Curr Microbiol 46, 345–352. 12732962 10.1007/s00284-002-3851-1

[25] Hooper NM (1994) Families of zinc metalloproteases. FEBS Lett 354, 1–6. 7957888 10.1016/0014-5793(94)01079-x

[26] Lacy DB and Stevens RC (1999) Sequence homology and structural analysis of the clostridial neurotoxins. J Mol Biol 291, 1091–1104. 10518945 10.1006/jmbi.1999.2945

[27] Montecucco C (1986) How do tetanus and botulinum toxins bind to neuronal membranes? Trends Biochem Sci 11, 314–317.

[28] Berntsson RPA , Peng L , Dong M and Stenmark P (2013) Structure of dual receptor binding to *botulinum* neurotoxin B. Nat Commun 4, 2058. 23807078 10.1038/ncomms3058 PMC3752466

[29] Rummel A (2017) Two feet on the membrane: uptake of clostridial neurotoxins In Uptake and Trafficking of Protein Toxins (Barth H, ed), pp. 1–37, Springer International Publishing, Cham, Switzerland. 10.1007/82_2016_48 27921176

[30] Rummel A , Eichner T , Weil T , Karnath T , Gutcaits A , Mahrhold S , Sandhoff K , Proia RL , Acharya KR , Bigalke H *et al* (2007) Identification of the protein receptor binding site of *botulinum* neurotoxins B and G proves the double‐receptor concept. Proc Natl Acad Sci USA 104, 359–364. 17185412 10.1073/pnas.0609713104 PMC1716154

[31] Strotmeier J , Mahrhold S , Krez N , Janzen C , Lou J , Marks JD , Binz T and Rummel A (2014) Identification of the synaptic vesicle glycoprotein 2 receptor binding site in *botulinum* neurotoxin A. FEBS Lett 588, 1087–1093. 24583011 10.1016/j.febslet.2014.02.034 PMC4067265

[32] Connan C and Popoff MR (2017) Uptake of clostridial neurotoxins into cells and dissemination In Uptake and Trafficking of Protein Toxins (Barth H, ed), pp. 1–40. Springer, Berlin Heidelberg. 10.1007/82_2017_50 28879524

[33] Lee K , Gu S , Jin L , Le TTN , Cheng LW , Strotmeier J , Kruel AM , Yao G , Perry K , Rummel A *et al* (2013) Structure of a Bimodular Botulinum neurotoxin complex provides insights into its oral toxicity. PLoS Pathog 9, e1003690. 24130488 10.1371/journal.ppat.1003690 PMC3795040

[34] Willems RJ , Top J , van Santen M , Robinson DA , Coque TM , Baquero F , Grundmann H and Bonten MJ (2005) Global spread of vancomycin‐resistant *Enterococcus faecium* from distinct nosocomial genetic complex. Emerg Infect Dis 11, 821–828. 15963275 10.3201/eid1106.041204 PMC3367597

[35] Natarajan P and Parani M (2015) First complete genome sequence of a probiotic *Enterococcus faecium* strain T‐110 and its comparative genome analysis with pathogenic and non‐pathogenic *Enterococcus faecium* genomes. J Genet Genomics 42, 43–46. 25619602 10.1016/j.jgg.2014.07.002

[36] Karlin S (2001) Detecting anomalous gene clusters and pathogenicity islands in diverse bacterial genomes. Trends Microbiol 9, 335–343. 11435108 10.1016/s0966-842x(01)02079-0

[37] Du P , Yang Y , Wang H , Liu D , Gao GF and Chen C (2011) A large scale comparative genomic analysis reveals insertion sites for newly acquired genomic islands in bacterial genomes. BMC Microbiol 11, 135. 21672261 10.1186/1471-2180-11-135 PMC3148964

[38] Moreno‐Letelier A , Olmedo‐Alvarez G , Eguiarte LE and Souza V (2012) Divergence and phylogeny of Firmicutes from the Cuatro Cienegas Basin, Mexico: a window to an ancient ocean. Astrobiology 12, 674–684. 22920517 10.1089/ast.2011.0685 PMC3426897

